# Pink1^−/−^ rats are a useful tool to study early Parkinson disease

**DOI:** 10.1093/braincomms/fcab077

**Published:** 2021-04-23

**Authors:** Cynthia A Kelm-Nelson, Sarah A Lechner, , Samantha E Lettenberger, Taylor A R Kaldenberg, , Natalie K Pahapill, , Amy Regenbaum, Michelle R Ciucci

**Affiliations:** Department of Surgery, Division of Otolaryngology-Head and Neck Surgery, University of Wisconsin Madison, Madison, WI 53706, USA

In the 2019 publication ‘To be or not to be pink (1): contradictory findings in an animal model for Parkinson disease’ by de Haas et al.,^1^ the authors presented important data with regard to the discrepancies in the number of substantia nigra dopamine neurons and extracellular striatal dopamine concentrations reported by their laboratory and others in the *Pink1*^−/−^ rat model of Parkinson disease. We agree with the data and the interpretation of the data with regard to this aspect of Parkinson disease. However, in their analysis, the authors excluded an important alternative perspective that Parkinson disease encompasses many other underlying mechanisms and deficits in addition to nigrostriatal dopamine depletion. Here, based on data from our laboratory and others, we propose that the *Pink1*^−/−^ genetic model is useful for studying signs and non-dopaminergic pathology, especially in this specific genetic rat model. Assessing if a model is useful based only on nigrostriatal dopamine mechanisms and associated behaviours ignores many other crucial aspects of Parkinson disease (discussed below) and can have serious negative consequences on advancing research and treatment approaches.

Parkinson disease has a complex pathology that is not well-understood. Parkinson disease is a progressive degenerative disorder that affects nearly 10 million people worldwide (1–2% of the population), and its incidence is expected to increase as the current population ages. It is commonly characterized and diagnosed by the following four major clinical signs: Bradykinesia, rigidity, tremors and postural instability. These motor signs are attributed to the substantial loss of the neurotransmitter dopamine. However, in most cases, neuron dysfunction and cell death (including outside the dopaminergic system) precede presentation of motor signs, likely manifesting at least a decade before a clinical diagnosis.^2^ Moreover, the mechanisms by which this occurs are currently unknown.

Parkinson disease encompasses pathology beyond nigrostriatal dopamine depletion. Parkinson disease pathology occurs at multiple levels throughout the central nervous system, including the lower brainstem, and affects multiple neurotransmitters and pathways. Additionally, current research has expanded our knowledge of underlying pathology to peripheral systems. For example, current hypotheses have focussed on the ascending spread of protein pathology (mainly aggregated α-synuclein) from the gut (via the vagus nerve) to the caudal brainstem. This is consistent with Braak central nervous system staging of Parkinson disease, where central nervous system pathology ascends in a caudal to rostral fashion as the disease progresses. Thus, early-stage (Braak Stage 1) Parkinson disease pathology is concentrated in brainstem regions such as the dorsal motor nucleus of the vagus nerve, anterior olfactory structures, and in Braak Stage 2 lower raphe nuclei and the locus coeruleus. This correlates with deficits in the noradrenergic system and early onset behavioural changes in vocalization, olfaction and attention. The substantia nigra is likely then impacted in Braak Stage 3; typically, this corresponds to the onset of the classical Parkinson disease motor signs and a clinical diagnosis. Again, this is consistent, as reviewed by Schapira et al., with the working hypothesis that Parkinson disease likely manifests years before significant nigrostriatal dopamine loss and the formal diagnosis.^2^

Importantly, just as in clinical neurology, some signs/symptoms (including non-motor features) were long overlooked, especially when they are not explained by the hallmark disease framework. Further, although a formal diagnosis relies on clinical motor features associated with dopamine depletion, there are other signs associated with earlier neurotransmitter changes. For example, dysautonomias are common including abnormal heart rate and blood pressure, REM behavioural sleep disorder, decreased saliva production, changes to swallow function and changes to gastrointestinal motility leading to constipation (reviewed in Metzger and Emborg^3^). Additionally, hyposmia linked to substance P and acetylcholine transmission has been noted. Early dysfunction within the noradrenergic system leads to sleep disruption, anxiety, depression and pain. Thus, the sole focus of dopamine depletion within the nigrostriatal frameworks ultimately limits our thinking of what Parkinson disease is and how we can effectively treat the disease. Below, we describe the usefulness of the *Pink1*^−/−^ rat model in studying early-onset Parkinson disease, non-limb motor clinical signs, and pathology outside of the classical nigrostriatal dopamine loss.

Genetic models of the disease can increase our general understanding of Parkinson disease. While nearly 80% of Parkinson disease cases are idiopathic, 5–10% are attributed to genetic mutations. Although known genetic cases occur less often, there is great utility in studying genetic forms of the disease. For example, proteins encoded by Parkinson disease-associated genes are involved in a set of molecular pathways that can trigger a pathology that is indistinguishable from sporadic forms. In addition, large genome-wide association studies show that these genes are also implicated in sporadic Parkinson disease. Early-onset Parkinson disease is inherently difficult to characterize and investigate in humans for a variety of reasons, including patterns of symptom manifestation, inconsistent age of disease onset and environmental variability. Primary advantages of a genetic model based on human inherited forms are homogeneity of the sample and better experimental control, which are significantly limited in human subject’s research. Thus, an excellent method to overcome these limitations *and* study this early phase of Parkinson disease as well as the associated pathological mechanisms, is *in vivo* genetic models. These models facilitate meaningful contributions to our understanding of Parkinson disease.

In humans, mutations in the PARK gene family cause protein accumulation in the brain that leads to neurodegeneration and are implicated in autosomal recessive, early-onset Parkinson disease. Like spontaneous forms of the disease, *Pink1* monogenetic variants [PTEN (phosphatase and tensin homologue)-induced kinase1; PARK6] demonstrate progressive deficits and nigrostriatal dopamine cell loss in the late stages of the disease, as well as other motor and non-motor deficits that may be related to non-dopaminergic mechanisms, e.g. early-onset bulbar sensorimotor deficits such as speech and swallowing dysfunction. These signs are clinically identical to those of individuals with sporadic forms of the disease. Similarly, the *Pink1*^−/−^ rat model demonstrates early motor and non-motor deficits and, as discussed in de Haas et al.,^1^ mild nigrostriatal dopamine loss at 8–10 months of age, providing a parallel link between the model and human Parkinson disease. Further, the 25–50% range of reported nigral dopamine loss in the *Pink1*^−/−^ rat model is consistent with Braak early-stage pathology.

*Pink1* ^−/−^ rats do show parallels to human Parkinson disease in terms of age of onset and progression of early cranial sensorimotor deficits. Bulbar-bilateral mediated motor acts and cranial-nerve mediated functions such as voice, speech and swallowing are substantially impaired in Parkinson disease, yet their actions are not fully linked to the classical Parkinson disease pathology. A major advance in our line of research is the use of the *Pink1*^−/−^ model for the examination of these cranial sensorimotor behaviours (e.g. ultrasonic vocalizations, tongue function, swallowing) along with physiological, morphological and biochemical measures including those within caudal brainstem regions and peripheral structures. This includes sex-specific differences that occur in Parkinson disease.^4^ Our laboratory has characterized cranial sensorimotor behaviour in the rat over the past decade; we have consistently reproduced these behavioural results. Our data have shown that the *Pink1*^−/−^ rat demonstrates significant and progressive deficits in vocalization and oropharyngeal swallowing as well as early-onset brainstem pathology and peripheral muscle alterations. For example, *Pink1*^−/−^ rats exhibit vocalization deficits beginning with reduced loudness at 2 months of age,^5^ an aspect of this model that has great translational relevance due to the nearly ubiquitous reduction in vocal loudness in human Parkinson disease. Vocal deficits then progress to reduction in quantity and quality of acoustic structure over time. In both humans and *Pink*1^−/−^ rats, these vocal changes appear earlier than limb-motor deficits and dopamine depletion. For example, we have shown that at 8 months of age, *Pink1*^−/−^ rats are slower and show more errors in the tapered balance beam test while vocalization deficits appear earlier.^5^ Additionally, vocalization deficits do not respond to levodopa and are likely related to significant neuropathological findings (e.g. aggregated α-synuclein) in the brainstem motor regions that control vocal output. The *Pink1*^−/−^ rat also demonstrates abnormal nociceptive responses and ventilatory abnormalities, suggesting that abnormal sensorimotor processing is occurring.^6^

We have also shown that the *Pink1*^−/−^ rat demonstrates swallowing impairments that are analogous to changes in human subjects.^7^ Within the brainstem, *Pink1*^−/−^ rats have 50% reductions in noradrenergic immunolabeled cells^5^ and significantly decreased norepinephrine protein concentrations in the locus coeruleus. Additionally, the relative mRNA expression of catechol-O-methyltransferase (*COMT*) is significantly upregulated in the locus coeruleus of *Pink1*^−/−^ rats, suggesting that increased presence of this enzyme is depleting the amount of norepinephrine in this region. We have also shown aggregated α-synuclein in brainstem motor nuclei. Further, our lab has investigated the neuromuscular properties of the thyroarytenoid (vocal fold) muscle and tongue muscles in the *Pink1*^−/−^ rat.^8^*Pink1*^−/−^ rats show increases in centralized nuclei, and changes to muscle fibres with decreases in muscle fibre size.^8^ Importantly, these muscular changes are negatively correlated to vocalization loudness, suggesting a deficit at the level of peripheral nervous system (unpublished lab data).

Finally, some of the most compelling evidence has been in our recent gene sequencing datasets, where we have shown that our large-scale gene analysis of *Pink1*^−/−^ tissue (brainstem) is matched to human idiopathic Parkinson disease and PARK6/2 gene databases.^9^ As such, our data and future work focus on the *Pink1*^−/−^ rat as a robust resource to (1) trace the complicated neuroanatomical pathways of vocalization; (2) identify the mechanism of disruption to vocal motor control; (3) test the application of pharmacological treatments in modulating cellular and behavioural outcomes. Our future work could provide further evidence that *Pink1*^−/−^ rats are a valid and natural translation to humans.

In general, *Pink1*^−/−^ rats provide us with the resource to test bulbar sensorimotor dysfunction. As a whole, the science and practice of neurology has historically ignored speech, swallowing and other signs of Parkinson disease when they do not fit within classical framework of nigrostriatal dopamine depletion. However, acknowledging these other aspects of Parkinson disease has opened doors to studying other pathological mechanisms, exponentially advancing our understanding of the disease. As such, we do not want to diminish the value of the *Pink1*^−/−^ model when there are robust deficits and mechanisms that parallel human Parkinson disease that are represented in this model, albeit sometimes imperfectly.

No genetic model of Parkinson disease is perfect, but the *Pink1*^−/−^ rat model is appropriate, for answering certain research questions. It is true that comparing animal models to Parkinson disease patients has its shortcomings because they are rodents and not humans.^10^ However, a major advantage of animal models, as well as cell culture, is that they allow thorough exploration of early physiological mechanisms. As such, animals do not have to be a perfect match to humans in order to serve as a *model* for disease. In fact, there are aspects of disease pathology that can be studied by multiple model systems to generate basic knowledge that is applicable to human patients. For example, collectively, genetic models like Pink1, SLC18A2, Ndufs4, ASO as well as neurotoxin models such as 6-OHDA cover the range of Parkinson disease signs. The view that a model needs to be perfect can obscure its usefulness, which ultimately limits research progress. We agree that this model may not be useful for studying nigrostriatal dopamine depletion, but that does not mean that model is not useful in many important ways. They key to using any model is to match the model to the research question and mechanisms of interest, discussed below.

It is important to match the model to the experimental question and to acknowledge limitations of each model. The *Pink1*^−/−^ rat model does have limitations, including less dopamine depletion than is seen in humans, hindlimb issues which may be an unknown consequence of the genetic mutation, and less behavioural robustness than the mouse model. As clearly described in de Haas et al.^1^, there have been studies by several groups, including ours, that have failed to replicate findings of consistent limb motor changes by 8 months of age with severity of nigrostriatal dopamine depletion. We agree with de Haas et al.^1^ that there is a lack of reproducibility of the original 25–50% reduction reported by Dave et al.^11^ Additional explanation for the discrepancies, not addressed in the paper, may include experimental controls such as light cycle, age and use of rat breeding lines, as well as social enrichment variables, especially with frequent behavioural testing. In fact, we found that when we do not assay multiple behaviours every 1–2 months, behavioural deficits and brain neurochemistry is different than in animals that receive consistent conspecific and experimenter/handler interactions.^12^ We feel that authors should make attempts to include these details in the experimental methodology sections of their manuscripts. The issue of genetic drift or decreases in strain quality have also not been addressed by the supplier; e.g. our group has noted inconsistencies in the presence of hindlimb dragging at 6 months among cohorts. Furthermore, there is a rat versus mouse limitation of the general *Pink1*^−/−^ model, as not all behavioural deficits and tissue changes manifest in the same way in other species. That being said, the rat may be more useful for complex behaviours and learning, and it is well-established for Parkinson disease behaviour.

The *Pink1*^−/−^ rat is a useful model to study Parkinson disease. The phenotypes of Parkinson disease are diverse in humans and animal models can only, at-best, partially mimic Parkinson disease. In essence, this is because is the very nature of Parkinson disease includes widespread and only partially understood pathology. However, at the behavioural level, *Pink1*^−/−^ rats tend to mimic the human symptomatology showing motor *and* also non-motor and non-classical motor impairments. As de Haas et al.^1^ conclude, the *Pink1*^−/−^ may not be the best model to study certain mid to late-stage characteristics of Parkinson disease, such as nigrostriatal dopamine depletion. However, our research demonstrates that the *Pink1*^−/−^ rat a valid model for testing a wide range of prodromal, early and progressive sensorimotor deficits including vocalization, tongue function (strength), chewing, swallowing, gait, spontaneous movement, fine motor control, anxiety, anhedonia, cognition and nociception as well as relevant underlying pathology such as noradrenergic dysfunction. Further, we have the opportunity to study brain−behaviour relationships at any of these early stages. Ultimately, the usefulness of a model depends on the research question. Importantly, these behavioural changes are beginning to be linked to biological changes outside of the classical nigrostriatal system. Thus, we believe that the *Pink1*^−/−^ rat is a useful model and should be continued to be used in research.

We thank you for considering these important points describing the usefulness of the *Pink1*^−/−^ rat model in our work.

## Data availability

Data sharing is not applicable to this article as no new data were created or analysed.

## Funding

This study was supported by National Institutes of Health, R01 DC014358, R01 DC019594 (M.R.C.) and R21 DC016135, R01 NS117469 (C.A.K.-N.).

## Competing interests

The authors report no competing interests.

## References

[1] de Haas R , Heltzel LCMW, Tax D, et al To be or not to be pink(1): Contradictory findings in an animal model for Parkinson’s disease. Brain Communications. 2019;1(1):fcz016. 31667474 10.1093/braincomms/fcz016 PMC6798789

[2] Schapira AHV , Chaudhuri KR, Jenner P. Non-motor features of Parkinson disease. Nat Rev Neurosci. 2017;18(7):435–450. 28592904 10.1038/nrn.2017.62

[3] Metzger JM , Emborg ME. Autonomic dysfunction in Parkinson disease and animal models. Clin Auton Res. 2019;29(4):397–414. 30604165 10.1007/s10286-018-00584-7 PMC6606399

[4] Marquis JM , Lettenberger SE, Kelm-Nelson CA. Early-onset Parkinsonian behaviors in female Pink1^−/−^ rats. Behav Brain Res. 2019; 377:112175. 10.1016/j.bbr.2019.112175 PMC6824965 31542395

[5] Grant LM , Kelm-Nelson CK, Hilby BL, et al Evidence for early and progressive ultrasonic vocalization and oromotor deficits in a PINK1 knockout rat model of Parkinson disease. J Neurosci Res. 2015;93(11):1713–1727. 26234713 10.1002/jnr.23625 PMC4575652

[6] Johnson RA , Kelm-Nelson CA, Ciucci MR. Changes to ventilation, vocalization, and thermal nociception in the Pink1-/- rat model of Parkinson's disease. J Parkinsons Dis. 2020;10(2):489–504. 32065805 10.3233/JPD-191853 PMC8142388

[7] Cullen KP , Grant LM, Kelm-Nelson CA, et al Pink1−/− rats show early-onset swallowing deficits and correlative brainstem pathology. Dysphagia. 2018;33(6):749–758. 29713896 10.1007/s00455-018-9896-5 PMC6207473

[8] Glass TJ , Kelm-Nelson CA, Russell JA, et al Laryngeal muscle biology in the Pink1-/- rat model of Parkinson disease. J Appl Physiol. 2019;126(5):1326–1334. 30844333 10.1152/japplphysiol.00557.2018 PMC6589810

[9] Kelm-Nelson CA , Gammie S. Gene expression within the periaqueductal gray is linked to vocal behavior and early-onset parkinsonism in Pink1 knockout rats. BMC Genomics. 2020;21(1):10.1186/s12864-020-07037-4 PMC7495669 32942992

[10] Chesselet MF , Carmichael ST. Animal models of neurological disorders. Neurotherapeutics. 2012;9(2):241–244. 22460561 10.1007/s13311-012-0118-9 PMC3337025

[11] Dave KD , De SS, Sheth NP, et al Phenotypic characterization of recessive gene knockout rat models of Parkinson's disease. Neurobiol Dis. 2014;70:190–203. 24969022 10.1016/j.nbd.2014.06.009

[12] Stevenson SA , Ciucci MR, Kelm-Nelson CA. Intervention changes acoustic peak frequency and mesolimbic neurochemistry in the Pink1-/- rat model of Parkinson disease. PLoS One. 2019;14(8):e0220734. 31374106 10.1371/journal.pone.0220734 PMC6677310

